# Predicting disease spread from host movement data: Chronic wasting disease in North America as a case study

**DOI:** 10.1111/1365-2656.70310

**Published:** 2026-07-22

**Authors:** Paul C. Cross, Blake Lowrey, Matthew Kauffman, Evelyn Merrill

**Affiliations:** ^1^ U.S. Geological Survey Northern Rocky Mountain Science Center Bozeman Montana USA; ^2^ U.S. Geological Survey, Wyoming Cooperative Fish and Wildlife Research Unit University of Wyoming Laramie Wyoming USA; ^3^ Department of Biological Sciences University of Alberta Edmonton Alberta Canada

**Keywords:** deer, ecological diffusion, invasion ecology, migration, spatial spread, wave speed, wildlife disease

## Abstract

1. Rare long‐distance movements can increase the spatial spread of invasive species and shifts in species ranges. For wildlife disease spread, however, seasonal migrations may only matter if they lead to transmission to individuals using different seasonal ranges, and the importance of juvenile dispersal depends on whether those juveniles are infected before they disperse.
2. High‐resolution animal movement data are increasing, but it is unclear how to leverage these data to improve predictions of future disease spread particularly in regions without pre‐existing movement data.
3. We predicted the speed of chronic wasting disease (CWD) spread based on movement metrics of mule deer (*Odocoileus hemionus*) and white‐tailed deer (*Odocoileus virginianus*) and compared our predictions to observed spreading rates in North America from 2005 to 2022. We hypothesized that CWD should spread faster in regions with long‐distance migratory mule deer, but this hypothesis was only partially supported.
4. Observed rates of CWD spread were faster in western epicentres with more mobile mule deer than in epicentres with only white‐tailed deer (14–18 km/year compared with 5–7 km/year, respectively). However, the observed speed of CWD spread did not align well with migration distances. Deer movements in the Canadian Prairie Provinces were far shorter than those in Colorado and Wyoming, yet Canadian Prairie Provinces had faster CWD spread, and, much of that spread was in open prairies where deer movements tend to be along riverine corridors rather than the longer elevational migrations in more montane regions.
5. Generally, our predicted rates of CWD spread from three different models were slower than the observed rates regardless of whether the model included dispersal or migration. Understanding these discrepancies is needed before high‐resolution animal movement data can be used to predict broad‐scale patterns of disease invasion.
6. Anthropogenic movements likely explain longer distance movements of CWD across North America, but it remains unclear whether human‐mediated movements or alternative analytical methods and deer movement metrics can explain the faster than predicted local CWD spread.

## Graphical abstract

This paper estimates the rate of chronic wasting disease spread in multiple regions and compares these rates with model predictions based on deer movement data.

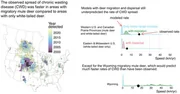

## 1 INTRODUCTION

There is a long history in ecology studying the spatial spread of mutations (Fisher, 1937), invasive species (Andow et al., 1990), and shifting species' ranges (Clark, 1998; Skellam, 1951). GPS technology has greatly expanded animal movement datasets, allowing detailed, high‐resolution documentation of animal movement and migratory corridors (e.g., Kauffman et al., 2020). As new diseases emerge, it will be tempting to use GPS movement datasets to predict the rate and routes of future disease spread. It is unclear which movement metrics (e.g. home range size, juvenile dispersal distances, or seasonal migrations) are the best predictors of pathogen spread in mobile wildlife, and the degree to which a tension exists between limiting pathogen spread and maintaining the connectivity of a host population (Mysterud et al., 2020). Previous research has highlighted the important role of rare, long‐distance movements, which may result in accelerating wave speeds (Clark, 1998; Mundt et al., 2009). However, limited work has been done using host movement data to predict the rate of future disease invasions, particularly at broad spatial scales. In this paper, we examined how predictions of disease spread based on movement metrics derived from GPS data compared with observed disease spread at regional and national levels. We use chronic wasting disease (CWD) as a case study, focusing on mule deer (*Odocoileus hemionus*) and white‐tailed deer (*Odocoileus virginianus*) movements in North America.

CWD is an infectious, neurodegenerative prion disease of cervids that was first recognized in Colorado in the 1960s (Williams & Young, 1980, 1993). As of May 2026, CWD has been detected in 36 U.S. States and 5 Canadian provinces in North America (National Academies of Sciences, Engineering and Medicine, 2024; Richards, 2021). CWD is fatal, without recovery, and is transmitted through both direct contact and indirectly through the environment (Miller & Williams, 2003). CWD spread likely results from a combination of natural deer movements that are influenced by landscape conditions as well as anthropogenic movement of deer among captive game farms, infected meat, body parts, cervid products, or contaminated feed or hay (National Academies of Sciences, Engineering and Medicine, 2024). Whether the multiple CWD epicentres across North America were the result of a single origin, or multiple independent events, is unknown and modelling the subsequent spread of CWD is hindered by the inability to apply traditional genetic approaches to this infectious protein. If CWD originated from a single event, natural deer movements alone seem unlikely to explain the disease's appearance in distant epicentres from the eastern United States to Alberta, Canada (Figure 1, Appendix S1).

**FIGURE 1:**
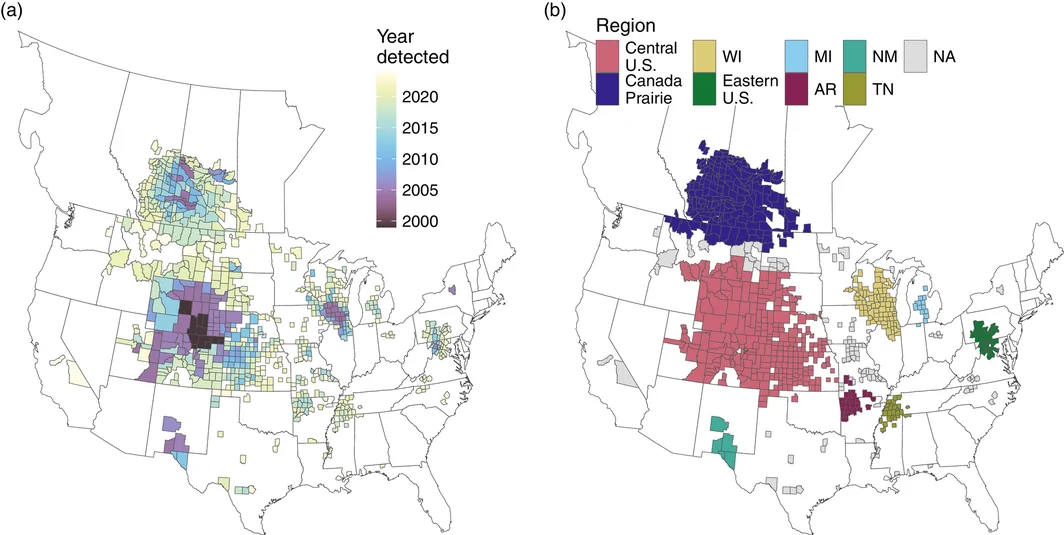
(a) Timing of first detections of chronic wasting disease (CWD) in different counties (United States) or Canadian Wildlife Management Units from 1999 to 2024. (b) Categorization of CWD into regional epidemics. Counties which were not contiguous with any epicentre or were consistent with multiple epicentres were not assigned (NA).

Previous CWD studies have focused on how patterns of disease distribution are related to host movement, habitat use, and contact rates (Barrile et al., 2024; Cullingham et al., 2011; Edmunds et al., 2017; Jennelle et al., 2022; Rogers et al., 2011; Walter et al., 2011), as well as work on landscape connectivity based on movements (Nobert et al., 2016; Russell et al., 2015) and genetic relatedness (Blanchong et al., 2008; Kelly et al., 2014; Powell et al., 2013; Robinson et al., 2013). These studies, however, fall short of making predictions of disease invasion speeds. Studies of disease data and pathogen genetics have estimated disease diffusion rates and inferred environmental covariates, but generally have not explicitly included host movement information (Biek et al., 2006, 2007; Hefley et al., 2017; Kamath et al., 2016; Kozakiewicz et al., 2018). Garlick et al. (2014), however, used GPS data from 22 mule deer to estimate CWD diffusion. They estimated wave speeds of 5–10 km/year depending on conditions, without the inclusion of migration or rare long‐distance movements. Here, we use deer dispersal and migration information to directly predict the rate of CWD spread across multiple epicentres and then validate (or contradict) those predictions using independent data on the observed rate of CWD spread.

Research on spreading populations traces back to population genetics (Dobzhansky & Wright, 1943; Fisher, 1937), with early ecological applications by Skellam (1951) and Kierstead and Slobodkin (1953). Contemporary work on invasion speeds has focused on invasive species and plant pathogens (Arim et al., 2006; Goldstein et al., 2019; Mundt et al., 2009), with few examples that connect GPS movement data to broad‐scale patterns of pathogen spread. Early models assumed movement could be approximated by a random diffusion coefficient, D, and did not account for rare, long‐distance movements. Empirical comparisons often have shown faster‐than‐predicted spread, attributed, in part, to unmeasured long‐distance dispersal (Allen et al., 1991; Clark, 1998; Dwyer et al., 1998; Lonsdale, 1993). Fat‐tailed dispersal kernels—those with higher probabilities of long‐distance movement than an exponential distribution—can lead to accelerating spread (Clark, 1998; Kot et al., 1996; Mollison, 1977), and previous studies of deer dispersal tend to show fat‐tailed dispersal kernels (Diefenbach et al., 2008). A further complication of adapting these models to wildlife disease is that animals generally remain in seasonal or permanent home ranges rather than continually diffusing across a landscape.

CWD was initially discovered around Colorado and Wyoming (Williams & Young, 1980) and Canadian investigations concluded that CWD may have been imported into Saskatchewan via captive cervids from the United States in the late 1980s (Kahn et al., 2004). The origins of the CWD epicentres in Figure 1 are unclear and surveillance was particularly limited prior to 2003, but documented commercial trade in farmed animals likely played a role (reviewed in National Academies of Sciences, Engineering and Medicine (2024)). CWD epicentres in the eastern and midwestern US are dominated by white‐tailed deer, which generally have limited migratory movements and small home ranges (<2 km^2^), but do have longer distance dispersal movements of juveniles (8–10 km on average; Diefenbach et al., 2008; Skuldt et al., 2010; Gilbertson et al., 2022). In contrast, CWD spread in areas with mule deer (western United States and Canada) may be driven by longer distance seasonal movements between winter to summer ranges that can be over 50–100 km (Kauffman et al., 2024).

Regional differences in host species, movement rates, densities, and general ecology prompt several hypotheses regarding CWD spread. One plausible prediction is that CWD may spread faster in regions with migratory mule deer populations (Conner & Miller, 2004). Disease speed, however, may be a function of both host movement rates and the rate of local disease transmission, whereby faster disease transmission could compensate for lower host movement rates. Both disease transmission and host movement are likely affected by habitat covariates and landscape connectivity (Nobert et al., 2016). Finally, juvenile deer dispersal could also be a mechanism for disease spread. The role of juvenile deer dispersal in CWD spread, however, is unclear because few juvenile deer are test‐positive for CWD (Heisey et al., 2010).

We compiled information from published studies, as well as GPS movement datasets, to compare deer movement and dispersal rates among regions with CWD. Where migration or dispersal data were available, we fit the empirical data to statistical distributions to assess the ‘fat‐tail’ nature of the movement kernels. We then used publicly available information on first detections of CWD in US counties and Canadian municipalities or wildlife management units (WMUs) to estimate the increasing area occupied by CWD, the associated wave speed, and any acceleration of CWD spread. We compared the observed wave speeds among regions and to predicted wave speeds based on movement metrics. Finally, we conducted sensitivity analyses to assess how our conclusions may be affected by delays in first detection due to the variable sampling for CWD in space and time.

## 2 METHODS

### 2.1 Deer movement

We gathered deer movement data from multiple regions, which can be large and contain varied ecosystems, herds, and species that did not always have a one‐to‐one correspondence with CWD epicentres. For the central United States, we used a combination of GPS data and literature‐based metrics from both Colorado and Wyoming. Specifically, we analysed GPS data from nine mule deer herds in Wyoming, USA (1423 animal‐years, Appendix S2; Kauffman et al., 2020; Kauffman et al., 2024; Russell, 2025) and also used the literature‐based metrics for mule deer in Colorado (Conner & Miller, 2004). In the Canadian Prairie Provinces (CPP) epicentre, we analysed mule deer and white‐tailed deer GPS datasets from Alberta (58 animal‐years for white‐tailed deer and 193 animal‐years of mule deer; Dobbin et al., 2023; Gritter et al., 2025) and data from the literature for Saskatchewan (Silbernagel, 2010; Skelton, 2010). Deer collaring efforts were covered by the following animal use and care protocols: Wyoming Game and Fish Department #22‐04, University of Alberta Animal Use and Care # AUP4641001, AUP00001369. For the midwestern and eastern US, we relied upon published dispersal data from white‐tailed deer in Pennsylvania (Jongejans et al., 2014; Long et al., 2008) and Wisconsin (Gilbertson et al., 2022; Table 1).

**TABLE 1:** Summary of chronic wasting disease (CWD) epicentres, data sources, and observed speed of CWD spread.

Epicentre	Host species^a^	Data sources			CWD		
		Migration	Dispersal	Home ranges	CWD timespan^b^	*r* ^c^	Speed^d^ (SD)
Eastern US	White‐tailed deer	NA	Literature	Literature	2007–2022	0.24	5.9 (6.9)
Wisconsin	white‐tailed deer	NA	Literature	Literature	2006–2022	[0.13–0.6]	6.5 (4.5)
Michigan	White‐tailed deer	NA	Literature	Literature	2017–2022	—	6.4 (14)
Central US	White‐tailed deer	—	—	—	2005–2022	[0.07–0.34]	14 (12)
Central US	Mule deer	This paper, literature	—	This paper			
CPP^e^	White‐tailed deer	This paper	—	This paper	2006–2022	[0.29–0.47]	18 (23)
CPP	Mule deer	This paper, literature	—	This paper, literature			

^a^ Elk and moose are also known hosts for CWD, but were not considered in these analyses.

^b^ Time span of the data used to estimate the observed CWD wave speed using the data from Richards (2021).

^c^ Annual growth rates of CWD based on Dugovich et al. (2024), Samuel (2023), Conner et al. (2021), Russell et al. (2026) and Smolko et al. (2021). Multiple values reflect the range of estimates across different subregions.

^d^ Observed CWD wave speed (km/year) for the listed time span.

^e^ Canadian Prairie Provinces of Alberta and Saskatchewan.

For the Wyoming and Alberta GPS data, we characterized distances between seasonal ranges, distances between consecutive winter ranges, and summer and winter home range area. We defined winter as 1 January through 31 March and summer as 1 July through 31 August and calculated the Euclidian distance between seasonal range centroids. We calculated the Euclidian distance between consecutive winter range centroids to characterize annual movements (Lowrey et al., 2020). We estimated seasonal range areas using the kernel density estimator in the *amt* package (Signer et al., 2019) in program R (R Core Team, 2024). To place our metrics of seasonal use on the same scale as our other movement metrics, we calculated the radius of the area within the 95th isopleth of the kernel density estimator. We censored animals with 10 or fewer locations in a season for all analyses (Appendix S2). Summarized movement distances and software code to replicate our analyses are available online (Cross & Lowrey, 2026).

To test the hypothesis that the spreading rate of CWD is associated with the frequency of rare long‐distance movements, we fit the dispersal and seasonal movement distances to multiple distributions with differing tail characteristics (Table 2). We used the *dispfit* package (Proença‐Ferreira et al., 2023) to fit the movement metrics to Gamma, Weibull, Log‐Normal, and Bivariate Student's *t* distributions. Some of the Wyoming mule deer herds were sampled more intensively than others (Appendix S2; Cross & Lowrey, 2026). In particular, the Red Desert herd had the most sampling at 718 individual years. We subsampled the Red Desert herd without replacement to include the same number of individuals as the next most intensively sampled region (*n* = 126). This constituted a compromise between reducing the dataset to the lowest common denominator of the most sparsely sampled region (*n* = 53) or allowing some regions to contribute far more individuals than others. We repeated the fitting process across 30 random subsamples of the Red Desert mule deer and report the average results.

### 2.2 Observed CWD spread

Richards (2021) compiled reports of new CWD detections in wild and game‐farmed deer across North America from 1999 to 2025. This dataset provides a coarse view of the expanding observed distribution of CWD but does not include sampling information. Schuler et al. (2024) compiled an alternative CWD database of annual prevalence and sampling intensity for 16 states in the Midwest and eastern United States. The Schuler et al. (2024) dataset was aggregated to the county‐level and individual data on sex, age, diagnostic test, and sample type (e.g., hunting, targeted cull, or vehicle accident) were not included. We compared the timing of first detections between these two datasets to assess their similarity where the two datasets overlapped, and we used the Schuler et al. (2024) data to evaluate CWD prevalence at the time of a county's first detection. The two datasets showed similar detection timelines, and Richards (2021) provided a broader spatial coverage that was not constrained by state boundaries, so we based our analyses of CWD spread on the Richards (2021) dataset.

The time‐lapse animated distribution of CWD (Appendix S1) illustrates that much of the CWD spread in wild deer within an epicentre has been neighbour‐to‐neighbour with only a small fraction of counties or WMUs that appear unassociated with a regional epicentre. Based on the early detections and subsequent spread of CWD, we defined eight regional epicentres: Central US (including Colorado, Wyoming, Utah, South Dakota, Nebraska, Kansas and parts of Montana), CPP (Alberta, Saskatchewan, Manitoba and parts of Montana), Wisconsin (including Illinois and parts of Minnesota, Iowa, and Missouri), Eastern US (including Pennsylvania, West Virginia, Maryland, and parts of Virginia), Michigan, Tennessee (including Mississippi), New Mexico and Arkansas (including parts of Missouri). We then assigned each newly detected county or WMU with CWD to these regional epicentres (Figure 1b). We assumed that counties or WMUs that were contiguous with other infected areas from 1 year before or after that detection were from the same epicentre. As epidemics from different regions merged into one another (Figure 1, Appendix S1), some counties and WMUs were difficult to assign unambiguously to a regional epicentre. In cases where the assignment was unclear, we left the county unassigned (Figure 1b), which was a conservative approach and leads to an underestimate of CWD wave speed.

Our original, less conservative, assignment of counties to a regional epicentre (Figure 1b) resulted in 9% of the total area being unassigned to an epicentre. Our more conservative definition resulted in 14.6% of the total area occupied by CWD being unassigned. We present the results of the less conservative assignment but note that the estimates of wave speed were not very different within a region when using the two approaches and did not change our overall conclusions (Appendix S1).

We estimated the observed rate of spatial spread using the total area occupied within each regional epicentre and across all regions. Specifically, we summed the area of those counties or WMUs with CWD and calculated the change in area occupied from 1 year to the next. To calculate the area of each county and management unit we used the NAD 1983 Albers Equal Area North America projection and the *sf* package in R version 4.4.0 (Pebesma & Bivand, 2023; R Core Team, 2024). County shapefiles were based on US Census Bureau TIGER data and the Canadian WMUs were downloaded from ArcGIS servers for each province (Alberta Government, 2024; Manitoba Government, 2024; Saskatchewan Ministry of Environment, 2024). We then approximated the wave speed as the annual change in the radius for circles of an equivalent area as the area occupied (Skellam, 1951). This process avoids issues with any discontinuities in the spatial distribution and averages over any anisotropic spread.

To statistically estimate the rate of observed CWD spread, we modelled the change in $\sqrt{\text{Area}}$ per year to avoid the autocorrelation associated with analysing cumulative area. Our statistical model for all epicentres combined was a traditional linear regression of the form:

$$\Delta \sqrt{\text{Area}}=\beta_{0}+\beta_{1}\times T+\epsilon$$

where ϵ is a normally distributed error term and T is the year scaled to be centred on the mean year (rounded down) for each epidemic. If the time span analysed was from 2005 to 2022, then *T* would range from −8 to 9. Dividing $\beta_{0}$ by $\sqrt{\pi }$ provides an estimate of the change in the radius, or wave speed, at the midpoint of the time span, and $\beta_{1}/\sqrt{\pi }$ relates to the acceleration, or deceleration, over time. For the regional analyses, we included a fixed effect for region and used a generalized linear model with a quasi‐Poisson log link function because the annual change was bounded by zero (Figure 2d). To test for differences among regions, we used the *ggeffects* package (Lüdecke, 2018). We excluded the Tennessee or Arkansas epicentres in the regional analyses due to the limited number of years prior to 2022 (3 or 5, respectively). We also did not analyse the regional spreading rate of the New Mexico epicentre given the limited spread recorded there (Figure 1).

**FIGURE 2:**
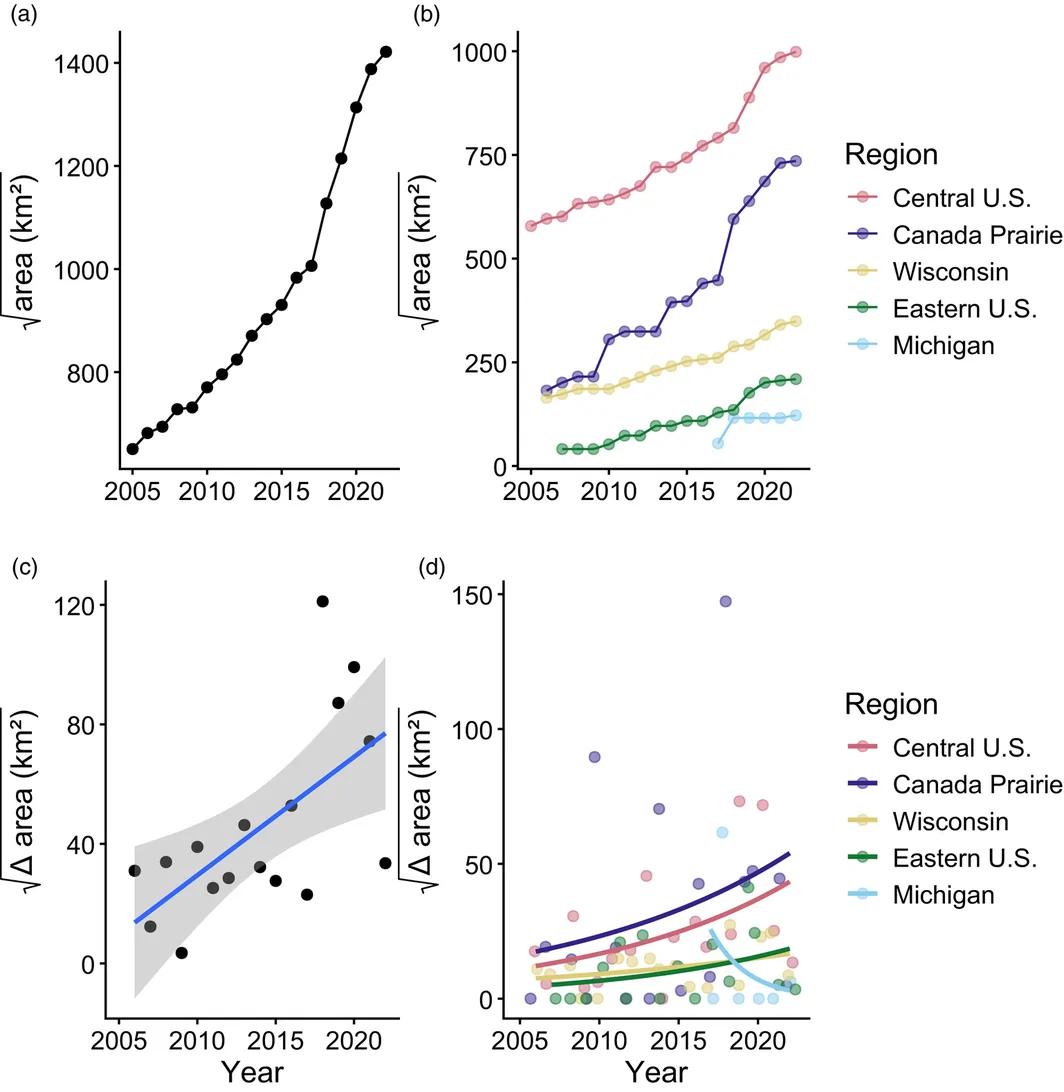
The square root of the area occupied by chronic wasting disease across North America from 2005 to 2022 (a) and by epicentre (b). The change in the square root of the area occupied overall with a linear regression (c) and by epicentre with a generalized linear model with a quasi‐Poisson log link function (d).

The initial detection of CWD is subject to sampling bias (Ruder et al., 2025). CWD sampling has varied over time and across jurisdictional boundaries. As a result, some regions may detect disease earlier or later than others for the same background prevalence. We addressed these biases through a variety of approaches. First, we excluded all data prior to 2005 because the maps were only updated annually from 2004 onwards, and we also excluded the first 2 years of data following the initial detection of CWD in a regional epicentre. Excluding these early years avoids some situations where a state or province may have limited initial sampling that rapidly expands after the initial detection to better characterize the full spatial extent of CWD in a new region. Second, we excluded data from 2022 onward because counties are increasingly difficult to assign to epicentres as they begin to merge (Figure 1). Third, we conducted sensitivity analyses of our observed spreading rates to additional time lags in detection at the county or state level. For the sensitivity analysis, we introduced additional detection lags into the dataset and then assessed how spreading rates and subsequent conclusions changed as a result (Appendix S3).

### 2.3 Predicted CWD spread

We compared the observed rates of CWD spread to predicted rates of spread based on deer movement metrics and temporal CWD growth rates. We used three different approaches to predict rates of CWD spread, which varied in whether they account for fat‐tailed movements or an attraction to a home range. First, we used the Fisher–Skellam approach, whereby the asymptotic rate of spread, *V*_FS_ = $2\sqrt{\mathrm{Dr},}$ where r is the instantaneous growth rate and *D* is the diffusion coefficient (Fisher, 1937; Kolmogorov et al., 1937). The Fisher–Skellam approximation assumes a Gaussian movement kernel and no home range attraction. Second, Jacobs and Sluckin (2015) provided simulated approximations of wave speeds for fat‐tailed movement distributions. We used their approximation of $V_{\mathrm{JS}}=0.71D^{0.64}$ for the stretched exponential movement distribution. Finally, Reluga et al. (2006) proposed a stochastic simulation model where infected individuals can move, but are constrained to a home range. We expanded upon their approach to allow a proportion of infected individuals to also either disperse, as in the case of Wisconsin and the Eastern US epicentres, or migrate, as in the case of the Central US and CPP epicentres. Our one‐dimensional simulation model accounted for fat‐tailed migration or dispersal movements as well as constrained movements in seasonal home ranges (Appendix S4).

For our simulations, we tracked the location of the furthest infected individual over time, and regressed the change in the furthest position, *x*, of an infected individual against time to estimate the speed and acceleration over a 20‐year timeframe. These simulations account for the possibility that some introductions of CWD into new areas will stochastically die‐out. In a subset of simulations, we allowed a fraction of individuals to disperse or migrate to a new home range. Dispersal distances were randomly sampled from Gamma or Log‐Normal distributions, depending on the region (Table 2, Table S4). We calculated the migration or dispersal distances moved in the *x* dimension as $d\cos\left(\theta \right)$ where *d* is the total movement distance and *θ* is a randomly chosen direction, in radians.

Deer movements are likely to vary within and across regions. We explored the differences between Wyoming and Colorado movements as well as between mule deer and white‐tailed deer in Alberta and Saskatchewan. To simulate Colorado and Saskatchewan migrations where we lacked raw data, we adjusted the fat‐tailed Gamma distribution to the reported mean but retained the shape parameter estimated from the Wyoming mule deer.

We compiled CWD growth rates, *r*, from the literature for our predictions of CWD wave speed (Conner et al., 2021; Dugovich et al., 2024; Samuel, 2023; Smolko et al., 2021). When annual CWD growth rates were not reported, we calculated *r* by rearranging the following relationship: $P_{\max}=P_{\min}e^{\mathrm{rt}}$ where $P_{\max}$ and $P_{\min}$ are the maximum and minimum prevalence, and *t* is the number of years between the two estimates (Table 1). We used prevalence estimates from periods in which CWD growth was approximately exponential based on visual assessment. To estimate diffusion coefficients, D, we divided mean squared movement distance by four (Case, 2000). We based our mean squared displacements on the migration distances described previously, dispersal distances reported in the literature, and seasonal range areas or home ranges from published sources. To convert home range areas to a distance metric, we calculated the radius of a circular home range with the same area as the 95th isopleth of the kernel density estimator.

Average dispersal and migration distances reported in the literature are sometimes limited to only juveniles or to those individuals that dispersed or migrated. For example, Gilbertson et al. (2022) estimated a dispersal probability of 18.8% across juvenile and adult white‐tailed deer in Wisconsin, whereas Long et al. (2005) estimated dispersal rates between 45% and 75% for juvenile white‐tailed deer in Pennsylvania. However, when adjusting for a stable age structure of approximately 26% juveniles (Jensen, 2000), the Long et al. (2005) dispersal rate would be approximately 16%, similar to Gilbertson et al. (2022). As a result, we used a 16% dispersal rate in our simulations. For epicentres with only white‐tailed deer (Eastern US and Wisconsin), we based the diffusion coefficient, *D*, on the weighted average of the 16% of the population that disperses and the home range radius of the remaining 84% of the population (Appendix S4, Table S4). For migratory mule deer populations, we assumed that all individuals had the possibility of migrating and thus used the distance between summer and winter seasonal ranges for all individuals as the ‘migration’ distance, even though in the case of resident deer, this distance may be small. In addition, some individuals may be infected on winter range, migrate and return to infect others on the same winter range the following year. We assumed that half of the transmissions occur in the same seasonal range as the infected individual was infected (e.g., on winter range) while the other half occur one migration away (e.g., on summer range).

## 3 RESULTS

### 3.1 Deer movement

As expected, we found that mule deer had high fidelity to the same winter ranges across years in both Wyoming, USA and Alberta, Canada (Appendix S4: Figure S6). Annual movement distances between winter centroids were on average 3.6 km in Wyoming (Appendix S2). Mule deer seasonal movements between winter and summer use areas averaged between 3 and 5 km in Alberta and from 15 to 117 km, across different Wyoming herds. White‐tailed deer in east central Alberta, Canada had similar movement distances compared with mule deer in that region (Appendix S2, Table S4), so we included the movement data from both species in our predictive models for Alberta. Conner and Miller (2004) provided migration distances for 12 mule deer herds in Colorado, with an average migration distance of 27.6 km from 117 migratory mule deer. However, 106 additional deer were considered resident with seasonal movements of around 2.6 km. Therefore, we used the weighted average movement distance of 15.7 km (Table S6) for our predictions of CWD wave speed. For the Saskatchewan deer, Skelton (2010) reported that 42% of deer migrated an average migration distance of 22.8 km. Assuming that non‐migrants move an average of 2 km (the radius of a 24 km^2^ home range; Silbernagel, 2010), the weighted average of migration distances would then be 11 km for mule deer in Saskatchewan.

Seasonal range metrics of deer in Wyoming and Alberta were right skewed, similar to the migration distances (Appendix S2, Figure 3). Five percent of the seasonal ranges were over 50 km^2^ for the 95th isopleth (Appendix S2, Figure S7). In most cases, this was due to our dates defining the summer and winter seasons that still captured some migratory movements, resulting in large seasonal ranges. As a result, we report the median and 90th percentiles for the home range metrics in Table S4. Most mule deer herds that we analysed had winter and summer home ranges with a radius of less than 3 km (Appendix S2, Table S4). The seasonal movements of Wyoming mule deer were best fit by a Gamma distribution, while deer movements in other regions were best fit by Log‐Normal distributions (as indicated by AIC, Table 2, Figure 3). In all cases, the best fitting distribution is considered fat‐tailed (Table 2), which suggests that CWD wave speeds might accelerate through time.

**FIGURE 3:**
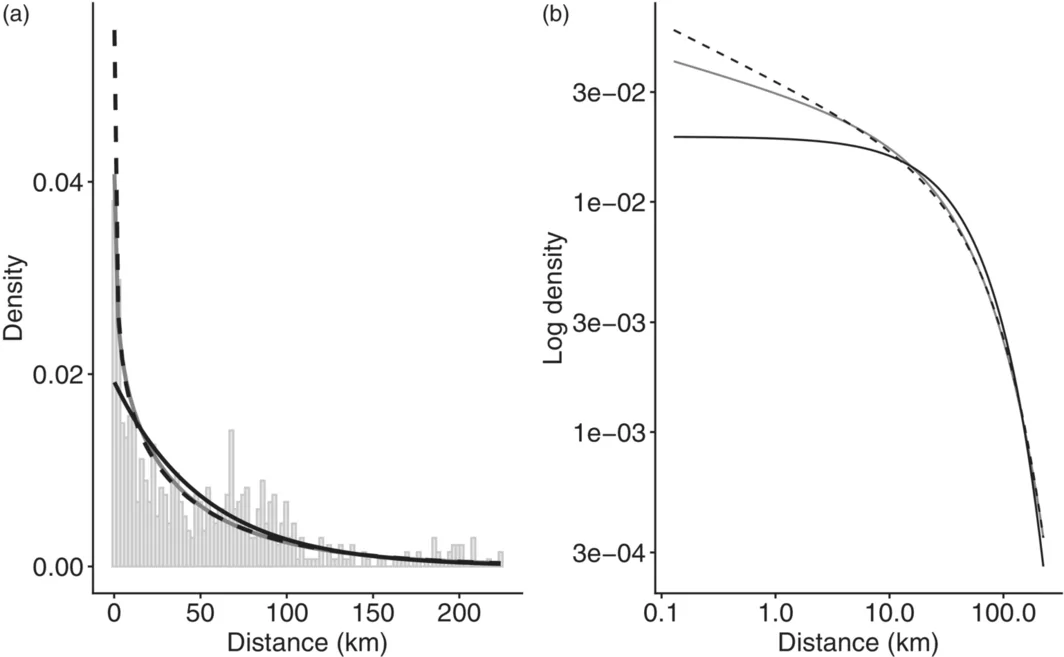
Distribution of winter‐to‐summer mule deer movements (km, *n* = 664) across seven herds in Wyoming on the natural (a) and log scales (b). The solid black line is the fitted exponential distribution. The dashed line is the Weibull distribution, and the solid grey line is the Gamma distribution.

**TABLE 2:** Parameter estimates and AIC values for statistical distributions fit to movement metrics from mule deer in Wyoming, mule deer and white‐tailed deer in Alberta, and white‐tailed deer in Wisconsin and Pennsylvania.

Metric	Distribution	Parameter *a*	95% CIs	Parameter *b*	95% CIs	Average	SD	AIC
Wyoming winter to summer movement								
Gamma		68	[60, 79]	0.76	[0.69, 0.84]	52	60	11,790
Weibull		49	[44, 54]	0.86	[0.81, 0.92]	52	61	11,803
Log‐Normal		24	[21, 27]	1.6	[1.5, 1.7]	89	324	11,965
2Dt		11	[7.9, 14]	1.4	[1.3, 1.5]	Inf	Inf	12,250
Alberta winter to summer movement								
Log‐Normal		1.8	[1.5, 2.2]	1.3	[1.1, 1.4]	4.1	8.2	1695
2Dt		0.8	[0.62, 1.1]	1.5	[1.4, 1.6]	Inf	Inf	1707
Weibull		3.5	[2.9, 4.3]	0.8	[0.68, 0.84]	4.1	5.5	1733
Gamma		6.2	[4.9, 8]	0.7	[0.59, 0.84]	4.4	5.2	1750
Wisconsin dispersal								
Log‐Normal		8.3	[7.3, 9.3]	0.7	[0.59, 0.76]	10	7.8	1675
2Dt		9.9	[7.4, 14]	2.4	[2, 3.1]	11	Inf	1684
Gamma		5.1	[3.9, 6.7]	2.1	[1.6, 2.6]	11	7.4	1701
Weibull		11.8	[10, 14]	1.3	[1.2, 1.5]	11	8.2	1715
Pennsylvania dispersal								
Log‐Normal		6.7	[6.2, 7.3]	0.6	[0.57, 0.68]	8.2	5.6	3012
Gamma		3.3	[2.7, 4]	2.5	[2.1, 3]	8.3	5.2	3046
Weibull		9.3	[8.5, 10]	1.5	[1.4, 1.6]	8.4	5.7	3075

*Note*: Parameters *a* and *b* relate to the scale and shape parameters, respectively, see table 1 of Proença‐Ferreira et al. (2023). Note that when parameter *b* of the Gamma and Weibull distribution is less than one, the resulting distribution is considered fat‐tailed. Algorithms could not converge for the Pennsylvania Bivariate Student's *t* distribution (2Dt).

Abbreviations: CIs, 95% confidence intervals; Inf, infinite as the mean cannot be calculated for all parameter ranges of some distributions.

### 3.2 Observed disease spread

The Richards (2021) and Schuler et al. (2024) CWD datasets showed reasonable concurrence in the timing of CWD detection for the 91 counties that were included in both datasets. Eighty‐eight percent of the matched counties were within 1 year for the recorded time of first detection. Only four counties differed by more than 3 years between the two datasets. We used the Schuler et al. (2024) dataset to assess potential detection lags as indicated by high prevalence at the time of first detection. We found that prevalence at the time of first detection was, on average, 1.8%, with 78% of the counties under 2%. Excluding Tennessee and Arkansas, which were not included in our subsequent analyses, the maximum prevalence at first detection was 5.8% with 95% of the counties under 4.3% prevalence.

The statistical regression of the change in the area occupied across epidemics in all regions from 2005 to 2022 (Figure 2) resulted in a $\beta_{0}$ of 41.4 (SE = 6.4), or a wave speed of 23.3 km/year (SE = 3.6). The change in radial speed significantly increased with time ($\beta_{1}$ = 3.96, SE = 1.3, *p*‐value = 0.01) suggesting that there was acceleration in the wave speed of about 2.2 km/year^2^ (SE = 0.72). This acceleration, however, was contingent on whether data on CWD in 2004 from the Central US epicentre was included in the analysis. Including 2004 data increased the midpoint spreading rate to 28 km/year (SE = 5.1), but the acceleration became non‐significant (0.94 km/year^2^, SE = 1.0) because a large increase in the observed area occupied occurred in 2005. When we analysed each epicentre separately, there was no significant acceleration of the wave speed for any epicentre (Figure 2d). The CWD wave speed was significantly faster in the CPP (18 km/year) compared with Michigan, Wisconsin, or the Eastern US epicentres (6.4, 6.5, and 5.6 km/year, respectively) based on pairwise comparisons (*p*‐value <0.5), but was not significantly faster than the Central US epicentre (Appendix S1, Tables S2 and S3). The CWD wave speeds in Michigan, Wisconsin, and the Eastern US epicentres were not significantly different from one another (Appendix S1, Tables S2 and S3).

Sensitivity analyses suggested that additional lags in CWD detections of 2–5 years on a proportion of counties or states had limited effects on our estimated CWD spreading rates (Appendix S3). Within a region, or across all the regions, the estimated wave speeds were only a few kilometres per year different (generally less than 3 km/year) under the different sensitivity analysis scenarios compared with our observed estimates (Appendix S3), which would not affect our overall conclusions.

#### 3.2.1 Predicted CWD spread

Our predictions of CWD spread were similar across the three different models when dispersal and migration movements were excluded (Table 3). Our simulation model, however, predicted faster spreading rates than the other models when including either dispersal or migration movements. The Eastern US and Wisconsin epicentres predictions were slower than CPP and Central US epicentres, similar to what was observed. The predicted rates, however, were generally slower than observed rates of spread (Tables 1 and 3, Figure 4). This discrepancy remained when we included the possibility of juvenile dispersal. Only when the CWD annual growth rate was elevated to a very high level (*r* = 0.6 from Samuel (2023)) did model simulations approach the observed speeds (Table 3, Appendix S4). Predictions of CWD wave speed in Wyoming were the exception; predictions using migration movements tended to exceed the observed rates of spread (Table 3, Figure 4). Wave speeds simulated for Alberta and Saskatchewan vastly underestimated the observed spread of 18 km/year (Table 3, Figure 4). Our simulations resulted in mostly linear rates of spatial spread, except for Alberta at very high CWD temporal growth rates of 0.6 (Figure S13).

**TABLE 3:** Summary of predicted chronic wasting disease (CWD) wave speeds across different regions with or without long‐distance dispersal or seasonal movements between winter and summer ranges. Model predictions were based on the parameter estimates in Table S4.

Dispersal or migration	Movement region	*V*_FS_ ^a^	*V*_FS_	*V*_JS_ ^b^	*V*_sim_ ^c^	*V*_sim_
		*r* = 0.3	*r* = 0.6	—	*r* = 0.3	*r* = 0.6
Migration	Wyoming	14.7	20.8	19.7	28 (15)	69 (21)
Migration	Colorado	4.7	6.7	4.6	8.1 (4.6)	20 (6.0)
Migration	Saskatchewan	3.7	5.3	3.4	5.9 (3.2)	14 (4.4)
Migration	Alberta	1.5	2.1	1.1	4.8 (4.2)	46 (24)
Dispersal	Wisconsin	1.2	1.7	0.8	3.2 (1.8)	9.7 (3.8)
Dispersal	Eastern US	1.1	1.6	0.7	2.2 (1.3)	6.5 (2.3)
None	Wyoming	0.88	1.2	0.5	0.65 (0.25)	1.5 (0.30)
None	Colorado	0.88	1.2	0.5	0.65 (0.24)	1.5 (0.30)
None	Saskatchewan	1.4	2.0	1.0	1.1 (0.41)	2.4 (0.50)
None	Alberta	0.82	1.2	0.5	0.64 (0.24)	1.4 (0.29)
None	Wisconsin	0.38	0.54	0.19	0.31 (0.11)	0.64 (0.13)
None	Eastern US	0.49	0.70	0.26	0.37 (0.14)	0.83 (0.17)

^a^ Fisher–Skellam velocity approximations of *V*_FS_ = 2 (*Dr*)^0.5^. The diffusion coefficient, *D*, was based on average home range sizes (no dispersal or migration) or was a weighted average that included the possibility of dispersal or seasonal migration.

^b^ Jacobs and Sluckin approximation of *V*_JS_ = 0.71*D* ^0.64^.

^c^ Predicted velocity from the simulation model with and without long‐distance movements averaged over 50 simulations (SD).

**FIGURE 4:**
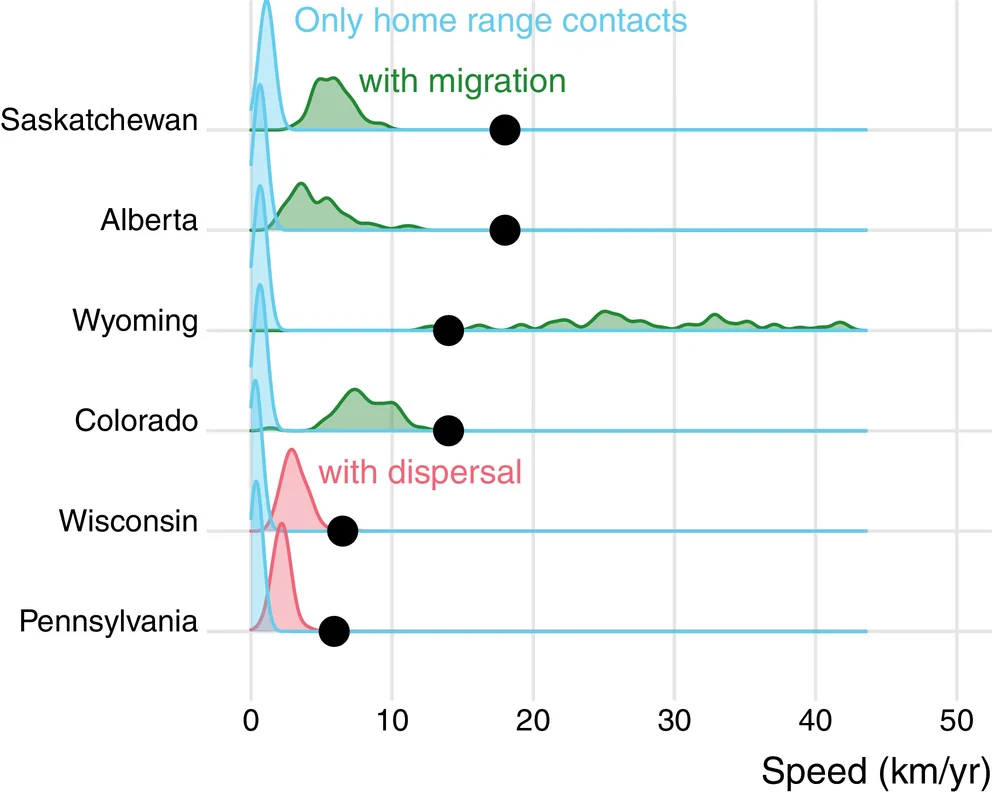
Density histograms of predicted chronic wasting disease (CWD) spread for 50 simulations using deer movement characteristics from five different areas assuming a CWD instantaneous growth rate of *r* = 0.3. Observed CWD wave speeds are represented by black points. Model simulations restricting individuals to only infect others within a home range are in light blue. Simulations that allow for fat‐tailed dispersal or migration distances are in pink and green, respectively.

## 4 DISCUSSION

This study was inspired by the work of Clark (1998), who questioned why trees migrate so quickly. Seed dispersal data combined with classical diffusion models could not explain the observed range expansions, and this juxtaposition of two different types of data, combined with model predictions, led to hypotheses about the role of extreme dispersal events. Despite the increasing availability of fine‐scale animal movement data, few studies have used that information to predict the diffusion rate of their associated pathogens, particularly at broad spatial scales (but see Garlick et al., 2014). From 2005 to 2022, CWD spread across North America at an average rate of 26 km/year (SD = 18), accelerating by 2.2 km/year^2^. The speed of CWD spread varied among regions, and was faster in epicentres in Central US (14 km/year) and in Canada Prairie Provinces (18 km/year) with mule deer compared with areas with only white‐tailed deer (Wisconsin = 6.5 km/year, Michigan = 6.4 km/year, and Eastern US = 5.9 km/year). Our predicted disease wave speeds were similar across multiple different models when we excluded deer migrations and juvenile dispersals. As expected, accounting for fat‐tailed movement kernels, juvenile dispersal and seasonal migrations resulted in faster disease spread. However, our model predictions tended to be several times slower than the observed rates even when dispersal and migration were included. This suggests that additional mechanisms of disease spread, such as anthropogenic movements, which were not included in our models, may be contributing to the regional spreading rates of CWD.

The role of wildlife movements in spreading disease can be case‐dependent and difficult to generalize to unsampled areas. For example, mule deer movement distances were far longer in Wyoming compared with Alberta or Saskatchewan, and yet the CPP had the fastest spread of CWD. In montane regions, ungulate migrations are driven by elevational movements to summer ranges. However, disease spread may depend on transmission between individuals from different winter ranges on the same summer range, or along migration corridors or stop‐over areas. For elevational migrations to drive disease spread a series of connected mountain ranges may be required. In contrast, in the CPP epicentre much of the observed spread is in agricultural and prairies regions where the many seasonal deer movements are not elevational but along riverine corridors and forest patches (Hefley et al., 2017). The role of juvenile dispersal in CWD spread, and many other diseases, is debatable because few juveniles test‐positive. Vertical transmission of CWD has been observed (Sandoval et al., 2025), but would be rare and hard to detect at low prevalence. However, even when we included juvenile dispersal, observed CWD speeds were faster in Wisconsin, Michigan, and Eastern US than what we predicted (Figure 4).

Local disease transmission rates are likely to correspond to faster wave speeds. We focused our results on a temporal growth rate, *r*, of 0.3, but explored growth rates up to 0.6 in Table 3 and Figure S14. A growth rate of 0.6 would result in predicted wave speeds that are more aligned with our observed rates (Table 3, Figure S14). However, *r* = 0.6 results in a CWD prevalence that increases from 2% to 22% in just 4 years. Samuel (2023) estimated that adult male white‐tailed deer had such high growth rates in a few regions, but these are likely extreme cases. Overall temporal trends in multiple studies suggest that growth rates are commonly less than 0.35 (Conner et al., 2021; Dugovich et al., 2024; Russell et al., 2026; Samuel, 2023; Smolko et al., 2021).

The vast distances between regional epicentres suggest an important role for anthropogenic movements seeding new outbreaks in disparate regions of North America (Makau et al., 2020; Rorres et al., 2018). For our analyses, a spillover from a game farm to wild deer would add only one additional county or WMU to the total area occupied and thus many spillover events from cervid farms would be required to substantially contribute to our estimate of spread. However, Richards (2021) notes numerous positive captive facilities in the fast spreading CPP epicentre (Appendix S1). The importance of other human‐mediated movement (e.g., carcasses), however, is unknown. Future research could estimate what disease dispersal kernel is required to create the observed pattern of CWD spread similar to Keeling et al. (2004). This movement kernel would be the combined effects of both natural and human movements.

Many states, provinces and countries have altered management strategies to control the spread and prevalence of CWD (Conner et al., 2021; Thompson et al., 2023; Whetten et al., 2025). Our high‐level analyses indicated relatively similar speeds among the epicentres with similar ecology and host species (e.g., Eastern US, Wisconsin and Michigan, compared with Central US and CPP). Analysis of the effectiveness of those management efforts in slowing spatial spread should account for sampling and management intensity (Whetten et al., 2025) as well as the potential for CWD‐induced declines to affect hunting as opposed to the reverse mechanism of hunting slowing the growth rate of CWD (Moss et al., 2025).

Previous authors have noted that the shifting sampling intensity makes it challenging to disentangle the effects of sampling and geographic spread (Evans et al., 2014; Ruder et al., 2025; Thompson et al., 2023). We share this concern, particularly when investigating CWD spread and directionality at finer spatial scales. Sampling variability in time and space may result in detection delays. However, so long as areas of infection are eventually discovered, this will appear in our analyses as increased variability, but similar average annual increases in the infected area (Figure 2c,d). Our sensitivity analyses of inducing longer detection delays to a subset of counties or states did not alter estimated wave speeds by more than a few kilometres per year (Appendix S3). Finally, we did not assess the role of different county or WMU sizes in our analyses. We expect that larger counties and WMUs result in larger, but less frequent, steps in the spread of disease compared with the same wave speed in a region with smaller counties.

Hefley et al. (2017), Xu et al. (2022), and Garlick et al. (2014) provide three other estimates and predictions of CWD spatial spread. Hefley et al. (2017) estimated a diffusion rate of 30–40 or 3.1–3.5 km/year in radial wave speed using disease data from Wisconsin. Xu et al. (2022) mechanistically predicted a spreading rate for Alberta to be around 6–7 km/year using movement information. Garlick et al. (2014) used deer movement data to predict CWD wave speeds of 5–10 km/year in Utah. All these studies underpredict the observed rate of CWD spread in their regions and did not address the potential role of rare long‐distance movements. Detailed models that account for how host movements change in different habitats are difficult to extrapolate to unsampled areas and computationally challenging to apply to broad national scales (but see Garlick et al., 2014).

This paper suggests that additional mechanisms may be playing a role in CWD spread beyond what we included here, or that the modelling approaches we used need to be further modified. By juxtaposing different datasets and model predictions we emphasize gaps in current understanding and future research needs. Many studies do not have an opportunity to be wrong because their findings are not compared with alternative data, models, or predictions; however, conflicting results reveal where existing theory and models fall short of explaining observed patterns. Additional successful case studies are needed before animal movement data can be used to confidently predict the rate and direction of pathogen spread.

## AUTHOR CONTRIBUTIONS

Paul C. Cross conceived the ideas and wrote the initial manuscript. Paul C. Cross analysed the data with assistance from Blake Lowrey on movement datasets. All authors contributed data and contributed to the conceptual development, manuscript editing, and presentation.

## CONFLICT OF INTEREST STATEMENT

The authors declare no conflicts of interest.

## Supporting information


**Appendix S1.** Animated map of the chronic wasting disease distribution in North America and alternative definitions of epicenters.
**Appendix S2.** Additional information on deer movement data.
**Appendix S3.** Sensitivity analyses of the observed CWD spread.
**Appendix S4.** Additional information on the simulation model and results.

## Data Availability

Data on movement distances, first observations of CWD, and simulation code are available here: https://doi.org/10.5066/P132GPWX Cross and Lowrey (2026).
