# Supplementary material for: Predicting disease spread from host movement data: Chronic wasting disease in North America as a case study
Source: J Anim Ecol. 2026 Jul 22;95(10):1919–32. doi: 10.1111/1365-2656.70310 (PMC13643186; doi:10.1111/1365-2656.70310)
Supplement: Supplementary file 1 [file JANE-95-1919-s001.zip › jane70310-sup-0001-AppendixS1-S4-FigureS1-S14-TableS1-S6@07_02_Appendices_clean.docx]

**Appendix S1: Animated map of the chronic wasting disease distribution in North America and alternative definitions of epicenters.**

The animation of the CWD areas (Figure S1) assisted in the assignment of counties and wildlife management units to regional epicenters. In this appendix, we investigated the impacts of those alternative definitions of regional epidemic membership (Table S1).

 ***Figure S1****. Animated map of chronic wasting disease distribution over time in United States counties and Canadian wildlife management units from 2007 to 2024 based on Richards (2021)*.

Not shown in the animated map (Figure S1) is that the Utah counties in 2004 were not contiguous with the epicenter of CWD in Colorado and Wyoming, creating some ambiguity as to whether Utah counties should be considered as part of the same epicenter. In the main text, we assumed that Utah counties were part of the Rocky Mountain region and in the more conservative definition (Figure S2) they were excluded from the analysis. In addition, some northern counties of Wisconsin coincided with detections in game farm detections (Figure S1) and were not contiguous with other counties with infected wild deer at that time. As a result, it is possible their origins may not have been limited to adjacent wild deer populations. These Utah and Wisconsin counties were not included in the more conservative assignment (Figure S2). We note, however, that a more conservative definition also potentially biases the regional rates of CWD spread to be low.

***Figure S2****. Two different definitions of regional membership, more conservative (A) and the definition used in the main text (B).*

***Figure S3****. The square root of the area occupied by chronic wasting disease across North America given the more conservative definition of regional membership from 2005 to 2022 (A) and by epicenter (B). The change in the square root of the area occupied overall with a linear regression (C) and by epicenter with generalized linear model with a quasi-poisson log link function (D).*

***Table S1.*** *CWD wave speed estimates under different definitions (in Fig. S2) and including or excluding 2004. Average and standard deviations of wave speeds (km/yr) were calculated from the raw data, while midpoints, accelerations, and their standard errors (km/yr^2^) were based on regression estimates, β_0_ and β_1_ respectively.*

| Metric | Definition | Years Included | Eastern U.S. | Wisconsin | Michigan | Central U.S. | Canadian prairie |
| --- | --- | --- | --- | --- | --- | --- | --- |
| Average speed (SD) | |  |  |  |  |  |  |
|  | Original | 2005-2022 | 5.9 (6.9) | 6.5 (4.5) | 6.4 (14) | 14 (12) | 18 (23) |
|  | Conservative | 2005-2022 | 5.9 (6.9) | 5.5 (3.1) | 6.4 (14) | 14 (12) | 18 (24) |
|  | Original | 2004-2022 | 5.9 (6.9) | 6.5 (4.5) | 6.4 (14) | 17 (19) | 18 (23) |
|  | Conservative | 2004-2022 | 5.9 (6.9) | 5.5 (3.1) | 6.4 (14) | 18 (20) | 18 (24) |
| Speed at midpoint (95 CI) | | |  |  |  |  |  |
|  | Original | 2005-2022 | 5.3 [2.8, 9.8] | 6.3 [4.6, 8.6] | 6.1 [1.1, 34] | 12 [7.9, 18] | 17 [9.4, 31] |
|  | Conservative | 2005-2022 | 5.3 [2.8, 9.8] | 5.4 [4.1, 7.1] | 6.1 [1.1, 34] | 12 [8.1, 18] | 16 [8.2, 32] |
|  | Original | 2004-2022 | 5.3 [2.8, 9.8] | 6.3 [4.6, 8.6] | 6.1 [1.1, 34] | 18 [11, 29] | 18 [9.4, 32] |
|  | Conservative | 2004-2022 | 5.3 [2.8, 9.8] | 5.4 [4.1, 7.1] | 6.1 [1.1, 34] | 18 [11, 30] | 16 [8.2, 32] |

The average rates of CWD spread within regions were not very different across the two regional definitions regardless of whether 2004 to 2005 data were included.

**Table S2.** Model results from the quasi-poisson generalized linear model of the observed change in the square-root of the area occupied by CWD from 2005 to 2022. Epicenters were included as fixed effects and using the epicenter definitions in Figure 1. Year was rescaled to be centered on the midpoint of each region. The estimated dispersion parameter was 22.2.

| **Parameter** | **estimate** | **SE** | **t-value** | **p-value** |
| --- | --- | --- | --- | --- |
| Intercept | 3.082 | 0.241 | 12.80 | 1.14E-19 |
| CPP^1^ | 0.345 | 0.306 | 1.13 | 0.264 |
| Michigan | -0.697 | 0.618 | -1.13 | 0.264 |
| Wisconsin | -0.695 | 0.409 | -1.70 | 0.094 |
| Eastern U.S. | -0.811 | 0.430 | -1.89 | 0.063 |
| Year (re-scaled) | 0.069 | 0.027 | 2.53 | 0.014 |

*^1^* CPP = Canadian Prairie Provinces

**Table S3.** Pairwise comparisons of epicenter contrasts for the model in Table S2, using the *ggeffects* package (Lüdecke 2018).

|  |  | **95 % CI** | |  |
| --- | --- | --- | --- | --- |
| **Comparison** | **Contrast** | **Lower** | **Higher** | **p-value** |
| CPP^1^ vs. Michigan | 20.4 | 2.74 | 38.2 | 0.024 |
| CPP vs Wisconsin | 20.4 | 5.94 | 34.9 | 0.006 |
| CPP vs Eastern U.S. | 21.7 | 7.33 | 36.0 | 0.003 |
| Central U.S vs. CPP | -9.2 | -25.2 | 6.8 | 0.259 |
| Central U.S. vs. Michigan | 11.2 | -5.02 | 27.5 | 0.175 |
| Central U.S. vs. Wisconsin | 11.2 | -1.43 | 23.9 | 0.082 |
| Central U.S. vs. Eastern U.S. | 12.4 | -0.014 | 24.9 | 0.050 |
| Michigan vs. Wisconsin | -0.017 | -14.5 | 14.5 | 0.998 |
| Michigan vs. Eastern U.S. | 1.2 | -13.2 | 15.6 | 0.869 |
| Wisconsin vs. Eastern U.S. | 1.2 | -8.99 | 11.4 | 0.814 |

*^1^* CPP = Canadian Prairie Provinces

**Appendix S2: Additional information on deer movement data**

GPS data from 9 mule deer herds in Wyoming were used to characterize seasonal home range sizes, movement distances between summer and winter ranges and annual movements between consecutive winter ranges (Figure S4, S6, S7, and Table S4).

***Figure S4.*** *Ninety percent minimum convex polygons of the mule deer herds from Wyoming.*

***Figure S5.*** *Ninety percent minimum convex polygons of the mule deer and white-tailed deer locations for the Canadian herds used in the Alberta analyses.*

Similar analyses were conducted on GPS data from white-tailed deer and mule deer in Alberta (Figure S5, S6, S7 and Table S4).

***Figure S6.*** *Distributions of mule deer movement distances between winter and summer ranges (A) and between winter ranges between years (B). Blue herds represent those from Wyoming, U.S.A. while red herds represent those from Alberta, Canada. Five deer from the Red Desert herd that moved 250-335km between winter and summer ranges were excluded from the plot.*

***Figure S7****. Distribution of winter and summer range sizes for mule deer herds in Wyoming, U.S.A. and Alberta, Canada. Distance (km) refers to the radius related to the area within the 95% isopleth of the kernel density. Blue herds represent those from Wyoming, while red herds represent those from Alberta.*

***Table S4:*** *Summary of deer movement metrics in Wyoming (WY) and Canadian Prairie Provinces (CPP). All deer were mule deer except for the 58 deer in the North Border study area that were white-tailed deer (WTD).*

|  |  |  |  | Winter to summer (km) | | |  | Winter to winter (km) | | |  | Winter use radius (km) | |  | Summer use radius (km) | |
| --- | --- | --- | --- | --- | --- | --- | --- | --- | --- | --- | --- | --- | --- | --- | --- | --- |
| CWD Epicenter | State/Province | Herd | Individual Years | Mean | SD | 90th |  | Mean | SD | 90th |  | Median | 90th |  | Median | 90th |
| Central United States | |  |  |  |  |  |  |  |  |  |  |  |  |  |  |  |
|  | WY | Lander | 81 | 15 | 14 | 35 |  | 2.0 | 2.1 | 4.8 |  | 1.7 | 3.8 |  | 0.79 | 2.6 |
|  | WY | Riverton | 126 | 16 | 27 | 51 |  | 5.0 | 6.3 | 13 |  | 1.8 | 3.8 |  | 1.2 | 2.2 |
|  | WY | Meeteetse | 87 | 28 | 21 | 64 |  | 2.3 | 4.6 | 4.4 |  | 1.5 | 2.6 |  | 0.95 | 1.6 |
|  | WY | Wind River | 110 | 29 | 33 | 92 |  | 1.2 | 1.0 | 2.4 |  | 1.7 | 2.8 |  | 1.5 | 4.1 |
|  | WY | Clarks Fork | 78 | 47 | 27 | 74 |  | 1.4 | 2.6 | 2.1 |  | 1.4 | 3.4 |  | 1.2 | 2.5 |
|  | WY | Dubois | 98 | 53 | 28 | 77 |  | 2.2 | 2.8 | 4.6 |  | 1.4 | 3.3 |  | 1.0 | 1.9 |
|  | WY | Upper Shoshone | 78 | 75 | 24 | 102 |  | 1.8 | 2.3 | 3.8 |  | 1.2 | 2.1 |  | 1.0 | 1.9 |
|  | WY | Owl Creeks | 53 | 89 | 29 | 126 |  | 6.7 | 20 | 6.6 |  | 1.4 | 2.2 |  | 1.2 | 5.3 |
|  | WY | Red Desert | 738 | 117 | 71 | 205 |  | 9.5 | 21 | 20 |  | 2.5 | 7.5 |  | 1.1 | 2.8 |
| Canadian Prairie Provinces | |  |  |  |  |  |  |  |  |  |  |  |  |  |  |  |
|  | Alberta | Wainwright | 35 | 3.2 | 2.2 | 5.2 |  | 1.2 | 2.2 | 1.7 |  | 1.4 | 2.9 |  | 1.3 | 2.5 |
|  | Alberta | North Border | 70 | 3.4 | 3.4 | 6.7 |  | 2.7 | 4.0 | 8.0 |  | 1.5 | 3.3 |  | 1.1 | 2.2 |
|  | Alberta | North Border (WTD) | 58 | 3.9 | 4.9 | 11 |  | 3.6 | 3.8 | 9.0 |  | 1.5 | 3.7 |  | 0.78 | 2.1 |
|  | Alberta | Cresthill | 88 | 5.5 | 13 | 13 |  | 1.3 | 0.63 | 1.9 |  | 1.5 | 2.3 |  | 1.3 | 2.5 |

*^a^* Distance between winter and summer centroids.

*^b^* Distance between the winter centroids across two consecutive years.

*^c^* Home range radius is the $\sqrt{{area}/\pi}$ , where the area is based on the 95^th^ isopleth of kernel density estimator for the season.

*^d^* 90^th^ percentile of the distribution.

*^e^* The North Border study area included both mule deer and white-tailed deer (WTD).

**Appendix S3: Sensitivity analyses of the observed CWD spread**

We conducted sensitivity analyses where we induced additional time lags to CWD detections at the county and state levels. We then assessed how spreading rates and subsequent conclusions changed as a result. We randomly chose 30 or 50 percent of counties and WMUs and further delayed their year of CWD detection by an additional two or five years and then conducted the statistical analyses of CWD spreading rates. We conducted the sensitivity analysis again by randomly selecting half of the states or provinces, then applied two-year or five-year detection lags to all counties or WMUs within those chosen areas. We conducted the state and province-level sensitivity analyses for the overall spreading rate across all epicenters or in the Central U.S. epicenter because other regional epicenters had too few states or provinces. We compared 100 simulations for each sensitivity analysis to the observed results. We removed the Michigan epicenter from the sensitivity analyses because it begins in 2017 and so including additional five-year lags would remove all those counties from the analysis because the last year of included data is 2022.

***Figure S8.*** *Estimated CWD wave speeds overall (A) and by epicenter (B) comparing 100 simulated datasets with an added 5-year lag to first detection in 30% of counties (histogram bars) to the observed estimates with no added lag (purple lines) based on data from 2005-2022. Acceleration estimates overall (C) and by epicenter (D).*

***Figure S9.*** *Sensitivity analyses assuming a 2-year additional lag to detection in 30 percent of counties. Purple lines represent the observed estimates of speed and acceleration, while the grey bars represent histograms of the 100 random simulations with additional lags to detection added to the dataset. A and B are the speed estimates overall and within each region, respectively, while C and D are the acceleration estimates.*

***Figure S10.*** *Sensitivity analyses assuming a 2-year additional lag to detection in 50 percent of counties. Purple lines represent the observed estimates of speed and acceleration, while the grey bars represent histograms of the 100 random simulations with additional lags to detection added to the dataset. A and B are the speed estimates overall and within each region, respectively, while C and D are the acceleration estimates.*

***Figure S11.*** *Sensitivity analyses assuming a 2-year additional lag to detection in 50 percent of states or provinces. Purple lines represent the observed estimates of speed and acceleration, while the grey bars represent histograms of the 100 random simulations with additional lags to detection added to the dataset. A and B are the speed estimates overall and the Central U.S. epicenter, respectively, while C and D are the acceleration estimates.*

***Figure S12.*** *Sensitivity analyses assuming a 5-year additional lag to detection in 50 percent of states or provinces. Purple lines represent the observed estimates of speed and acceleration, while the grey bars represent histograms of the 100 random simulations with additional lags to detection added to the dataset. A and B are the speed estimates overall and within each region, respectively, while C and D are the acceleration estimates.*

**Appendix S4. Additional information on the simulation model and results.**

We used three different models to predict CWD spread: the Fisher-Skellam model, the Jacobs and Sluckin (2015) approximation of their stochastic model, and a simulation model based on Reluga et al. (2006, Table S5). For each model, where possible, we compared the results that included or excluded longer-distance movements (either dispersal or seasonal movements). Model predictions were based on deer movement data from six regions (Table S6) that related to the different CWD epicenters (Figure 1). Our simulation and the Fisher-Skellam models allowed for different CWD growth rate parameters. We highlight results using a CWD growth rate of *r* = 0.3 as a commonly observed rate, but we include results up to *r*=0.6 to cover the observed range of possibilities even though *r* = 0.6 may be much above the average rate (Table 1).

**Table S5**. Description of different models used to predict the rate of disease spread.

| **Model** | **Movement metric^1^** | **Fat-tail^2^** | **Home range attraction^3^** | **Disease growth rate** | **Reference** |
| --- | --- | --- | --- | --- | --- |
| Fisher-Skellam | diffusion coefficient *D* | no | no | included | Fisher 1937 and Skellam 1951 |
| Jacobs & Sluckin | diffusion coefficient *D* | yes | no | not included | Jacobs & Sluckin 2015 |
| This paper | Home range radius | no | yes | included | modified from Reluga *et al*. 2006 |
| This paper | Home range radius and long-distance movements based on a Gamma or Log-Normal distribution | yes | yes | included | modified from Reluga *et al.* 2006 |

1. Eastern U.S. and Wisconsin epicenters use dispersal distances weighted by the proportion of juveniles, while non-dispersing individuals have a movement distance associated with their home range radius. Central U.S. and Canada prairie epicenters use migration distances weighted by the proportion migrating and resident animals have a movement distance associated with their home range radius.

2. Whether the model accounts for a fat-tailed or non-Gaussian movement distribution.

3. Whether the model accounts for an attraction towards a home range centroid or assumes memory-less diffusion.

*Simulation model*

We developed a simulation model adapted from Reluga *et al.* (2006) that allowed for both local contacts within a restricted home range as well as longer distance movements that would relate to either seasonal movements between winter and summer ranges or juvenile dispersal. For the simulation, we assumed the deer population is large and uniformly distributed over a one-dimensional space. Every individual has a spatial location, $x$, and a home range center, $x_{h}$. Regula *et al.* (2006) modeled a range of host-pathogen combinations, accounting for scenarios where the disease transmission was fast relative to host movement. In these cases, hosts may not have enough time between infection and being infectious to reach some spatial equilibrium within their home range. For CWD, there is likely a lag of several months between infection and being infectious, which is plenty of time for deer to re-distribute across their home range. The Regula *et al.* (2006) model then simplifies to assuming individuals’ location at time *t* can be approximated by a Gaussian distribution around their home range centroid.

$$p\left( x|x_{h},t=\infty\right)=\frac{1}{\sqrt{2\pi\delta/\alpha}}\exp\left[ \frac{-\alpha\left( x-x_{h} \right)^{2}}{2\delta} \right]$$

Where $\delta$ is some diffusion rate, not to be confused with the diffusion coefficient *D*, and *α* represents attraction to the centroid. We assume $\alpha=1$ so then $\delta$ becomes the variance of the home range distribution. We estimated $\delta$ using the 95% kernel density of empirical home ranges and the fact that 95% of a Gaussian distribution is within a distance of $\pm1.96\delta$.

We assume a fraction of infected individuals may either move between seasonal ranges or disperse from the location where they were initially infected. For Wisconsin and Eastern U.S. simulations we used the dispersal data of Gilbertson *et al.* (2022) or Long *et al.* (2008), respectively, while accounting for the smaller fraction of juveniles that disperse. For Colorado, Wyoming, Alberta, and Saskatchewan simulations we used migration distances while accounting for the fraction of individuals that may not migrate. For either dispersal or seasonal movements we randomly sampled movement distances from the distributions in Table S6. For Colorado simulations, we adjusted the scale parameter to result in a mean migration distance of 15.7 km (Conner and Miller 2004), but retained the shape parameter estimated from our Wyoming GPS dataset. We assumed that infected and susceptible individuals move similarly and ignored environmental transmission, so that transmission of infection occurs among individuals at the same position $x$. We assume individuals are removed at rate $\mu=0.5$ for an average infectious lifespan of 2 years. Each infected individual infects others at a rate of $\beta$.

Our recipe for the simulation follows that of Regula et al (2006). Suppose there are currently $C\left( T \right)$ infected individuals enumerated $j$ = 1,2,…$C\left( T \right)$ at time $T$ and the individual $j$ has a home range of $x_{h}\left( j \right)$, position $x\left( j \right)$ at some time $t\left( j \right)<=T$. Using the Gillespie algorithm we then randomly choose an individual $j$ and wait a time $\hat{t}=min\left( \hat{t}_{\beta},\hat{t}_{\mu} \right)$, where the time until the next transmission event is $\hat{t}_{\beta}\sim Exp\left( \frac{1}{\beta C\left( T \right)} \right)$ and the time to the next removal event is $\hat{t}_{\mu}\sim Exp\left( \frac{1}{\mu C\left( T \right)} \right)$. If the next event is a transmission, individual *j*’s current position $\hat{x}\sim f\left( x_{h}\left( j \right) \right)$ at time $T+\hat{t}$, where the function $f$ is a Normal distribution with a mean of $x_{h}\left( j \right)$ and variance of $\delta$ if the individual did not disperse. A fraction, $\rho$, of transmission events result from dispersals outside of the seasonal home-range. Dispersing individuals move a $z$ distance from their home range, where $z$ is randomly sampled from a Log-Normal distribution. For movements between seasonal ranges, we assume that half of the disease transmissions occur in a seasonal range that is one migration away from their home range centroid, while the other half are within the same home range as they were infected. The migration distance is then sampled from the distribution noted in Table S6. Simulated dispersal and migration distances had to be modified from Table S6 to account for the one-dimensional model since only a fraction of the total movement distance relates to movement in the *x* direction. Specifically, for each migration of dispersal movement we choose a random angle for that movement and the movement distance is then$z^{'}=z*\cos\left( \theta\right)$, where *θ* is the angle in radians.

Newly infected individuals have the same position $\hat{x}$ as the individual that infected them, but their home range centroid is $\hat{x}_{h}\sim N\left( \hat{x},\delta\right)$. The simulation then repeats for a newly chosen individual and time period. We monitored the position of the furthest-forward individual for a period of 20 years. All simulations started with ten infected individuals to minimize the possibility of early extinctions. We conducted a statistical analysis to estimate the overall speed and acceleration over the 20-year simulation. The statistical analyses were similar to the empirical data analysis whereby the change in the maximum *x* was regressed against year, such that the regression coefficient on year reflects any acceleration over time. We coded the model in R and C++ using R package Rcpp (Eddelbuettel et al. 2025). Initial models used the Gillespie algorithm described above, but the large numbers of infected individuals in some runs required us to convert the model to a fixed time interval of 0.1 year per timestep where the number of events per timestep was approximated by a Poisson distribution and mortality occurred prior to disease transmission. Model checks showed minimal differences between the two modeling approaches.

**Table S6.** Summary of parameters used to estimate wave speeds for five different regions

|  |  |  |  | Diffusion coefficient ^d^ | |  |  | Parameter | |
| --- | --- | --- | --- | --- | --- | --- | --- | --- | --- |
| Region | Migration^a^ | Dispersal^b^ | Range radius^c^ | migration or dispersal | home ranges | Distribution^e^ | *δ*^f^ | *a* | *b* |
| Colorado^g^ | 15.7 | -- | 1.6 | 19 | 0.64 | Gamma | 0.67 | 20.5 | 0.76 |
| Wyoming | 52 | -- | 1.6 | 180 | 0.64 | Gamma | 0.67 | 68.5 | 0.76 |
| Alberta | 4.1 | -- | 1.5 | 2.0 | 0.56 | Log-Normal | 0.59 | 0.62 | 1.27 |
| Saskatchewan^h^ | 11 | 10 | 2.6 | 12 | 1.69 | Gamma | 1.76 | 14.5 | 0.76 |
| Eastern U.S.^i^ | -- | 8.2 | 0.9 | 1.1 | 0.20 | Log-Normal | 0.21 | 1.9 | 0.62 |
| Wisconsin^j^ | -- | 10 | 0.7 | 1.2 | 0.12 | Log-Normal | 0.13 | 2.1 | 0.67 |

^a^ Mean migration distances accounting for the fraction of individuals that did not migrate.

^b^ Mean dispersal distances.

^c^ Radius of the home-range.

^d^ The diffusion coefficient, *D*, used for the *V*_FS_ and *V*_JS_ predictions. *D* was based on the migration distances for Canada prairies, Colorado and Wyoming. *D* was based on the average of the dispersal distance and the range radius weighted by the overall percentage of individuals dispersing, which was assumed to be 0.16 to account for age structure. For migratory populations, we weighted D by assuming that 50% of transmissions occurred one migration away from the seasonal home range where the individual was infected and 50% of transmissions occurred within the same home range where the individual was infected.

^e^ Distribution used to simulate either migration or dispersal distances using parameters *a* and *b* estimated from *dispfit* (Proença-Ferreira et al. 2023).

^f^ Variance of the Gaussian distribution used in the simulation of an individual’s home range

^g^ Colorado migrations were weighted by the fraction of individuals that did not migrate. Shape of the migration distribution was based on the shape of the Wyoming distribution, but scaled to the mean migration distances from Conner and Miller (2004).

^h^ Migration and dispersal distances were taken from Skelton (2010) and Silbernagel (2010). We used the migration distances for the velocity predictions.

^i^ We used the dispersal distances and rates from Long et al. (2008) in Pennsylvania and adjusted for a stable age distribution of 26 % juveniles.

^j^ We used the dispersal distances from Gilbertson et al. (2022) in Wisconsin.

***Figure S13.*** *Forward positions of a CWD outbreak for 50 simulations in different regions for disease transmission values that correspond to r = [0.1, 0.3, 0.6].*

***Figure S14.*** *Density histograms of predicted CWD spread for 50 simulations using deer movement characteristics from five different areas assuming a CWD instantaneous growth rates of r = [0.1, 0.3, 0.6]. Observed CWD wave speeds are represented by black points. Model simulations restricting individuals to only infect others within a home range are in light blue. Simulations with Gaussian dispersal kernels are dark blue. Simulations that allow for fat-tailed dispersal or migration distances are in pink and green, respectively. Wave speeds for Wyoming with r = 0.6 with migration exceeded 50km/yr and are excluded from the plot.*

**References**

Conner, M. M., & Miller, M. W. (2004). Movement patterns and spatial epidemiology of a prion disease in mule deer population units. *Ecological Applications, 14*, 1870-1881. <https://doi.org/10.1890/03-5309>

Eddelbuettel, D., Francois, R., Allaire, J., Ushey, K., Kou, Q., Russell, N., Ucar, I., Bates, D., & Chambers, J. (2025). Rcpp: Seamless R and C++ Integration. <https://doi.org/10.32614/CRAN.package.Rcpp>

Gilbertson, M. L. J., Ketz, A. C., Hunsaker, M., Jarosinski, D., Ellarson, W., Walsh, D. P., Storm, D. J., & Turner, W. C. (2022). Agricultural land use shapes dispersal in white-tailed deer (*Odocoileus virginianus*). *Movement Ecology*, *10*, 43. <https://doi.org/10.1186/s40462-022-00342-5>

Long, E. S., Diefenbach, D. R., Rosenberry, C. S., & Wallingford, B. D. (2008). Multiple proximate and ultimate causes of natal dispersal in white-tailed deer. *Behavioral Ecology,* *19*, 1235-1242. <https://doi.org/10.1093/beheco/arn082>

Lüdecke, D. 2018. ggeffects: Tidy Data Frames of Marginal Effects from Regression Models. *Journal of Open Source Software, 3*, 772. <https://doi.org/10.21105/joss.00772>

Proença-Ferreira, A., Borda-de-Água, L., Porto, M., Mira, A., Moreira, F., & Pita, R. (2023). *dispfit*: An R package to estimate species dispersal kernels. *Ecological Informatics,* *75,* <https://doi.org/10.1016/j.ecoinf.2023.102018>

Reluga, T. C., Medlock, J., & Galvani, A. P. (2006). A model of spatial epidemic spread when individuals move within overlapping home ranges. *Bulletin of Mathematical Biology,* *68*, 401-416. <https://doi.org/10.1007/s11538-005-9027-y>

Richards, B. J. (2021). Chronic wasting disease distribution in the United States by state and county (ver. 2.0, April 2024). *U.S. Geological Survey data release*. <https://doi.org/10.5066/P9HQKKFO>

Silbernagel, E. R. (2010). Factors affecting movement patterns of mule deer (*Odocoileus hemionus*) in southern Saskatchewan: Implications for chronic wasting disease spread. Masters thesis. University of Saskatchewan, Saskatoon.

Skelton, N. K. (2010). Migration, dispersal, and survival patterns of mule deer (*Odocoileus hemionus*) in a chronic wasting disease-endemic area of southern Saskatchewan. Masters thesis. University of Saskatchewan, Saskatoon.
